# Experimental Study on Warpage Phenomenon of Wax Parts Manufactured by Fused Filament Fabrication

**DOI:** 10.3390/polym16020208

**Published:** 2024-01-11

**Authors:** Muslim Mukhtarkhanov, Essam Shehab, Md. Hazrat Ali

**Affiliations:** Department of Mechanical and Aerospace Engineering, SEDS, Nazarbayev University, Astana 010000, Kazakhstan; muslim.mukhtarkhanov@nu.edu.kz (M.M.); essam.shehab@nu.edu.kz (E.S.)

**Keywords:** warpage, wax, FFF, rheometer

## Abstract

Warpage is one of the prominent issues in Fused Filament Fabrication. The cause of this is the rapid cooling of the polymer during extrusion. The residual thermal stresses accumulated within the print part result in a shape distortion and subsequent detachment of the object from the print bed. In this study, both experimental and numerical approaches were used to identify the stresses due to thermal shrinking that occurs in soft polymers such as wax. A temperature sweep test was performed using a rotational rheometer to measure the magnitude of axial forces that are generated due to the thermal shrinking of a thin layer of 3D printable wax. The thermal stresses responsible for warpage were computed analytically and using the FEA. It was found that due to thermal processes, the stress magnitude can reach a value of 1.17 MPa. This value is enough to cause the plastic deformation in the wax part having a thin elongated shape. In addition, Taguchi’s robust design has identified two major FFF parameters that impact the warpage in amorphous soft polymers. They are the printing speed and the print bed temperature. To achieve a low level of warpage, it is important to make sure that the layer deposition occurs at medium speeds and the print bed temperature is moderately high according to the findings of this study.

## 1. Introduction

Rapid investment casting (RIC) is the metal manufacturing process where sacrificial patterns of wax or non-wax nature are fabricated through rapid prototyping (RP). This results in reduced production costs due to the elimination of the expenditure spent on designing and manufacturing metal tooling. Over the last three decades, numerous RP methods have been tested for application in RIC [1]. Although it has been shown that most additive manufacturing (AM) technologies are suitable for rapid casting applications, not many techniques can manufacture wax patterns with properties similar to investment casting (IC) wax. As for the non-wax patterns, the quality of printed parts is much superior in comparison to wax patterns. Moreover, the cost of non-wax polymers is substantially lower. However, when it comes to the quality of final cast metal products, the usage of wax patterns yields higher-quality objects. That is because the tough non-wax polymers are difficult to remove from the ceramic mold due to the large thermal expansion of the former. Therefore, the material market for the AM industry has been constantly growing with the introduction of new filament materials that have qualities tailored to the needs of end users.

Among modern RP techniques, three types specialize in manufacturing wax patterns. They are MultiJet Printing (MJP), stereolithography (SLA), and Fused Filament Fabrication (FFF). Even though the technologies are based on layer-by-layer patterns of manufacturing, they are distinguished by the level of accuracy, production speed, and cost. For example, the MJP technology is capable of building objects with layers having a thickness as low as 16 µm in the z-build axis. This implies that high accuracy and detail are possible to achieve [2]. In 2021, Chyuan et al. [3] compared the dimensional accuracies of wax patterns manufactured using MJP and FFF-based rapid tooling. They concluded that the direct manufacturing of wax patterns using MJP technology is costly and, therefore, less expensive manufacturing routes can be selected with the help of rapid tooling. Nevertheless, it should be noted that the implementation of rapid tooling increases the level of complexity since additional processes are included in the sequence of manufacturing.

As for the SLA method, it uses photocurable resins as raw material, which can be rapidly cured with the help of a laser. The technology also produces highly detailed objects with good accuracy though parts with overhanging sections require supplementary structures called support structures. The removal of support structures is carried out manually which often leaves visible marks on the surface of the product. Due to the high cost of commercially available SLA-based castable wax, the production of small-size parts is preferable [4]. With regards to material properties, a photocurable wax formulation requires a burnout of the pattern as opposed to dewaxing which is commonly used in conventional IC manufacturing [5]. Moreover, the selection of the burnout schedule for the SLA castable wax is a nontrivial task since the material decomposes in a non-combustive manner. Thus, if small-size patterns are not difficult to thermally deteriorate [6], working with medium to large-size objects is associated with challenges [7].

**Figure 1:**
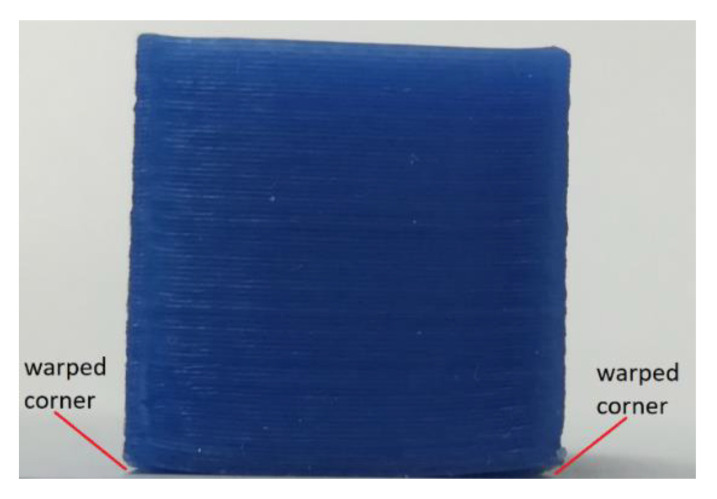
3D printed cube of 20 × 20 × 20 mm dimension.

The FFF technology employs a rather simple manufacturing process based on the extrusion of molten polymer through a heated small nozzle and depositing it on the print bed. Because the technique is unsophisticated, the quality of the final parts is inferior to MJP and SLA-printed objects. On the other hand, the affordability and availability of FFF machines make the technology the most popular in the AM industry. In addition, recent studies demonstrated that it is possible to manufacture low-cost wax patterns having properties very similar to traditional IC waxes [8,9]. For example, non-tough wax parts having low viscosity in the molten state have been manufactured by the authors of the current study [8]. Nevertheless, 3D printing of wax parts is associated with some challenges. For instance, during the extrusion process, the plastic polymer being deposited undergoes rapid solidification, creating thermal stresses within the body of the part. Those stresses manifest in shrinkage or shape distortion in the final product. In some extreme cases, the accumulated thermal stresses cause the part to detach from the print bed before the 3D printing is over. In this regard, mechanically weak polymers such as wax are more susceptible to warpage due to their inability to withstand large shrinkage stresses. This is visible in Figure 1 where the 3D printed wax part is shown.

Substantial efforts have been put into investigating the issue of warping in AM. Process parameters, material properties, and geometric characteristics are all said to influence the magnitude of the warpage [10]. In addition, numerical and experimental approaches have been proposed to analyze the warping phenomenon. The most common way to measure the warpage magnitude is realized through the evaluation of dimensional deviations in 3D printed benchmarks that represent simple geometric shapes. Table 1 shows the recent works related to the warpage phenomenon relevant to FFF. From Table 1 it is evident that warpage is mostly affected by several process parameters with layer thickness being the most frequently discussed parameter. It is interesting to note that some level of contradiction exists regarding the effect of layer thickness. For instance, while the majority of the publications support the view that a higher level of thickness is preferable to avoid warpage, the works of Antonio et al. [11] and Zhang et al. [12] contradict this view.

Setting up the higher layer thickness is beneficial for warpage mitigation according to the supporters of this view [13,15]. For instance, it is stated that at a lower thickness of the layer, the less mass of the material is deposited and, consequently, a layer of the extrudate cools at a faster rate [18]. In turn, the rapidly cooling polymer melt has little time to adhere to the print bed surface at the point of deposition. In contrast, the supporters of the opposite view have pointed out that physical mechanisms responsible for this phenomenon are yet to be discussed in the future [11].

The analysis of recent works shows that most of the studies concern the segment of materials representing hard polymers of both amorphous and crystalline kinds. For example, Liu et al. [15] have stressed the substantial work dedicated to amorphous ABS plastics regarding the warpage problem. As for semicrystalline polymers, there is an ongoing search to improve the printability of such plastics since the warpage level is critical in those materials and it is directly proportional to the degree of crystallinity [19]. In turn, the degree of crystallinity is the major factor affecting the mechanical properties.

While it is evident that for hard 3D printing polymers, the subject matter of warpage is largely explored, the issue can be considered unexplored for soft amorphous polymers such as wax. Therefore, the authors of this study find it extremely important to analyze the problem of warpage in soft 3D printable polymers. Especially, for materials intended for RIC application, where the control of dimensional accuracy gains even more significance. In essence, the current study is a continuation of a research project intended to explore the area of 3D printing wax polymers having low melting temperatures for application in RIC. Previously, research has been conducted by the authors to analyze the mechanical, thermal, and rheological properties of 3D printable wax [8]. In turn, the main objective of this paper is to address the prominent issue regarding wax 3D printing, which is warpage. To identify the FFF process parameters that cause the warpage, the design of experiments is performed using Taguchi robust design. In addition, rheological experiments are proposed to assess the shrinkage forces that develop during the rapid solidification of soft 3D printable wax.

## 2. Methodology

### 2.1. Materials and Characterization

Commercially available Ø1.75 mm 3D printing wax filament from Filamentarno (Moscow, Russia) in the blue color was purchased for the study. It has a melting temperature of around 100 °C and Young’s modulus of 151.5 MPa identified by tensile test [8].

### 2.2. Assessment of Shrinkage Forces Due to Rapid Cooling Using a Rheometer

**Figure 2:**
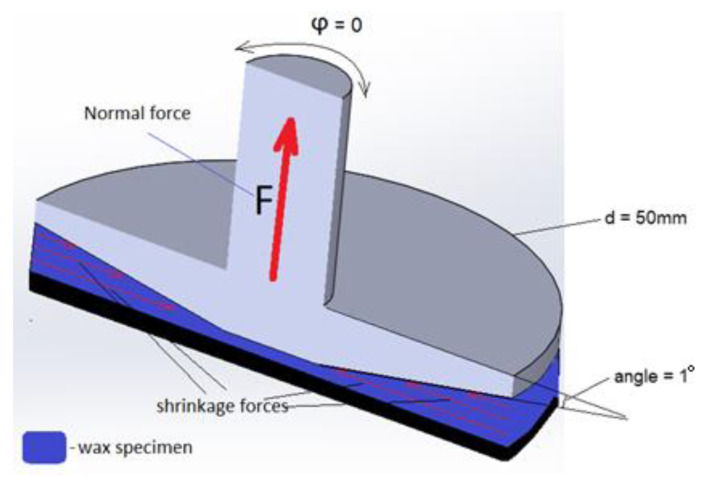
Vertical cross-section view of the cone/plate system with red arrows on the sample representing the forces that occur because of thermal shrinkage.

Rheological studies have been extensively used to characterize 3D printing polymers [20]. As for this study, we propose a temperature sweep test to understand the shrinkage behavior of the thin wax layer during solidification. A rotational rheometer MCR102 from Anton Paar (Graz, Austria) is used in the experiment with a cone and plate measuring system having Ø50 mm and a truncate angle of 1° as shown in Figure 2. The temperature sweep test allows the measurement of several important characteristics during the temperature change in a polymer sample confined between measuring system plates. Relevant to this study, we are interested in measuring the normal forces that develop in the upper plate of the rheometer as a response to the volumetric shrinkage of wax. It was observed that during the small amplitude oscillatory shear test, a noticeable increase in normal force is detected that acts on the measuring plates as wax transforms from liquid to solid state. This is illustrated in Figure 2 where shrinkage forces are represented as red horizontal arrows pointing towards the center of the cone plate. During the thermal shrinkage of the polymer, friction forces occur between the plate and the material as the latter tries to shrink. Insofar as the gap between plates remains unchanged, the deformation of the wax is suppressed. As a result, the force balance is created between the shrinking wax and the measuring system of the rheometer provided that the plates are under no oscillatory motion. Similarly, during the 3D printing process, a size decrease of the final part evidences the occurrence of thermal shrinkage. Usually, the first layer being deposited adheres to the print bed firmly, provided that the latter is hot enough. As the FFF continues, additional layers are added to the previously deposited layers. Since each individual layer is subject to shrinkage, the thermal stresses accumulate within the body of the solidified part. It is critical to understand that the shrinkage stresses act parallel to the deposition direction. Thus, the elongated objects are more prone to size contraction.

During testing, small granules of wax filament are placed on the lower plate of the equipment. Then, the plate is heated up to 110 °C to melt the material. After melting the sample, the upper plate is closed to reach the gap of 0.5 mm between the plates. To attain thermal equilibrium, the upper plate is preheated with the help of a heating hood accessory. Once sufficient time is given to reach thermal equilibrium, the temperature is lowered to 90 °C. The lower limit of 90 °C is chosen because the wax has a dropping point at 95 °C, thus, the material turns from liquid to solid state within the given temperature range. Moreover, to prevent an excess in normal force, it was decided to limit the lower value at 90 °C level. It should be noted that the measuring plates were subject to no oscillatory or rotational motion throughout the test. Since the sample has low thickness, the plain strain and plain stress conditions apply.

### 2.3. Design of Experiments for Identification of FFF Parameters Causing Warpage

**Figure 3:**
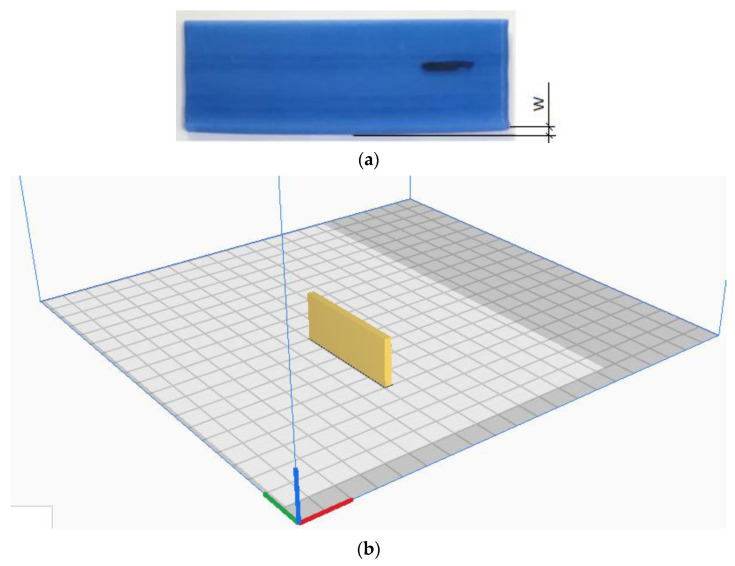
A benchmark for the warpage analysis (**a**); the position of the part on the print bed (**b**).

To analyze the effect of FFF process parameters on warpage occurrence, different kinds of experimental designs have been proposed previously [21]. Taguchi’s design of experiment (DoE) is one of the popular techniques that is broadly used to identify optimal process conditions for different manufacturing methods. An orthogonal array (OA) method was used to identify the effects of three FFF parameters on the level of warpage of a 3D printed part having the shape of a flat bar. Namely, the temperatures of the nozzle and print bed along with printing speed are selected for the experiment. The part has dimensions of 60 × 5 × 20 mm (L × W × H) and the 3D printed sample is shown in Figure 3.

The combinations of three FFF parameters at different levels require nine experiments according to L9 OA Taguchi analysis requirements. To increase the confidence in the results of the tests, each experiment is repeated three times, and an average value is used for calculations. By finding the signal-to-noise (S/N) ratio, it is possible to see the influence level of each parameter. Since warpage is mostly due to thermal processes, two out of three selected parameters relate to the temperature of the extrudate and the platform upon which the material is deposited. As for the printing speed, it influences the rate of solidification and interlayer bonding. Table 2 shows the process parameter values at different levels used in Taguchi analysis.

**Figure 4:**
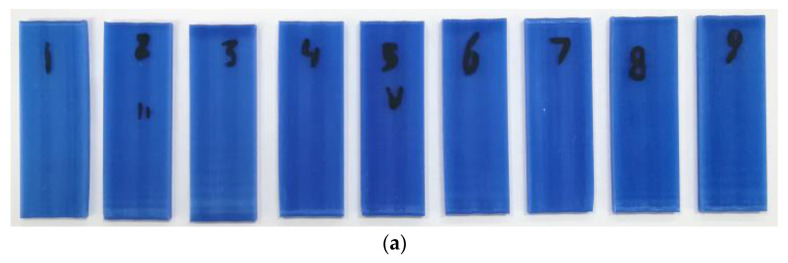

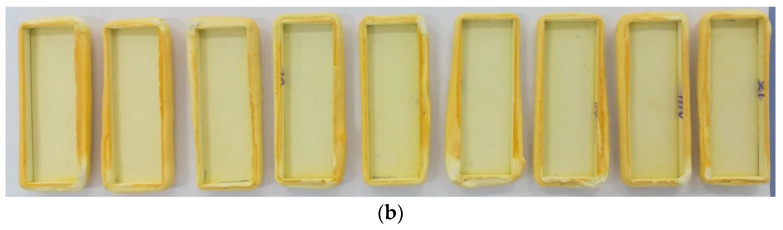
3D printed wax benchmarks for the Taguchi analysis (**a**); ceramic molds fabricated using the wax benchmarks (**b**).

Minitab 19 software was used to perform Taguchi analysis calculations. To measure the warpage magnitude W indicated in Figure 3a, the coordinate measuring machine (CMM) Duramax from ZEISS (Zeiss Group, Oberkochen, Germany) was used. The CMM machine uses a stylus system to scan the model, therefore, challenges arise when working with soft materials such as wax. To tackle the problem, the warpage level was measured on the ceramic molds shown in Figure 4b instead, which represent a complete replica of the artifact.

The ceramic shell was created on the surface of wax bars by dipping the latter in the ceramic slurry that is used in investment casting manufacturing. The wax bars and their corresponding ceramic molds are shown in Figure 4. Four layers of ceramic material were enough to build a strong shell with the help of silica-based ceramic slurry Suspendaslurry^®^ FS from Ransom & Randolf (Maumee, OH, USA). After the application of one layer, the parts are left to dry for three hours. To prevent the emergence of drying cracks, each layer was reinforced with a fused silica mesh 50/100. After the ceramic shell construction was completed, the wax models were manually removed to reveal the negative imprint of the benchmark.

**Figure 5:**
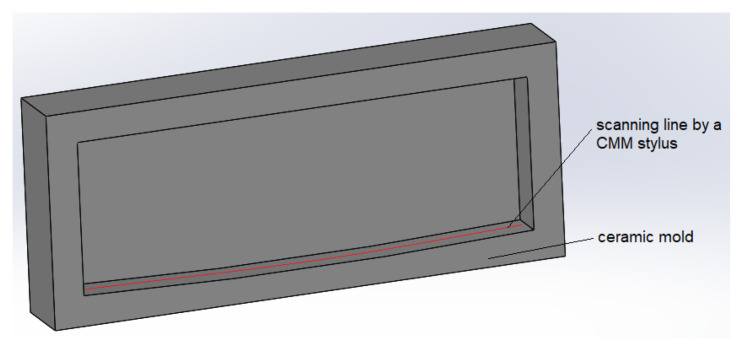
A location where measurements were performed by the CMM measuring tool.

The warpage magnitude is measured as the cartesian distance between the lowest and the highest point that lies on a curved line scanned from the warped plane as shown in Figure 5.

## 3. Results and Discussion

### 3.1. Identified Stresses Due to Thermal Shrinkage

**Figure 6:**
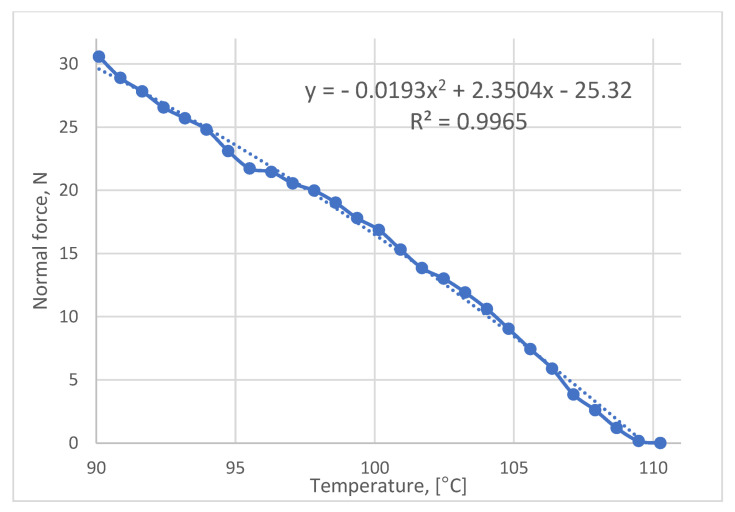
Normal force vs. temperature diagram as per rheometer test.

The result of the temperature sweep test is shown in Figure 6. The maximum developed normal force within the temperature range of 90–110 °C was measured to be equal to 30.57 N. The cooling rate was identified to be approximately 7.7 °C/min.

**Figure 7:**
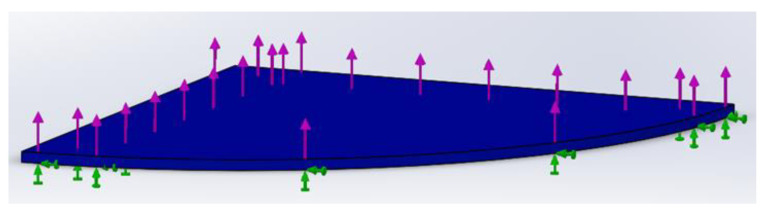
Boundary conditions for the FEA.

After finding the magnitude of the normal force caused by the shrinkage of wax, it is possible to evaluate the stress magnitude within the body of the wax sample. To accomplish the task, a Finite Element Analysis (FEA) can be used to implement the static structural module of the SolidWorks 2016 Simulation software. The boundary conditions for the static structural analysis can be established as follows. The wax sample in the form of a thin film having a circular shape with a diameter of 50 mm and thickness of 0.5 mm is subjected to a normal force of 30.57 N. This force is derived from the actual experiment described earlier and its direction coincides with the one identified experimentally. It should be noted that due to the circular shape of the model, it is symmetrical and, thus, for the FEA only a quarter of the model was considered as shown in Figure 7 and the magnitude of applied normal force was reduced accordingly. The wax sample is represented with a blue color and a fixed support indicated with green arrows was applied to the bottom side of the film. The purple arrows shown in Figure 7 represent the normal force. It should be noted that despite the plate having a conical shape, it is assumed flat due to a small truncate angle (1°). It should be noted that additional assumptions were made to simplify the analysis:

(a) The thermal effects including polymer relaxation were neglected. During solidification of the polymer, the mechanical properties change significantly. Although this type of transformation is neglected, it is assumed that all the stresses due to the shrinkage of the material accumulate as residual stresses in the solidified body.
(b) The polymer is assumed to have isotropic properties.
(c) During the FFF, the material is deposited in a layer-by-layer fashion. However, in FEA the entire body of thin film is assumed to be shrinking at once. Since it is observed that the warpage manifests with some delay in time for 3D printed parts, the stress accumulation within the entire body is likely the major factor for warpage occurrence.

**Figure 8:**
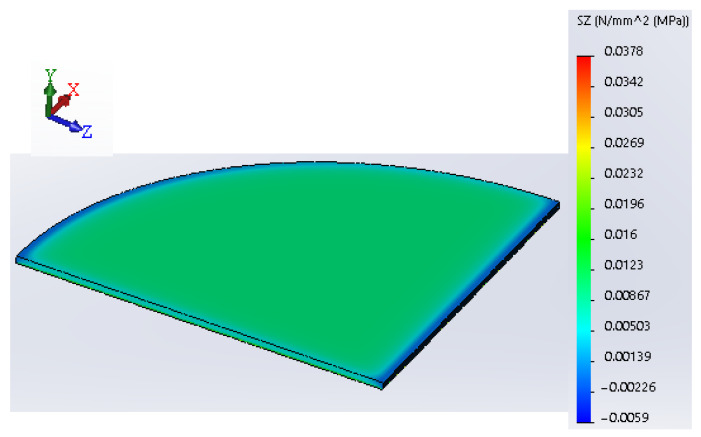
FEA results show a stress component in the lateral direction (load = 30.57 N).

A young’s modulus of 151.5 MPa and Poisson’s ratio equal to 0.4 were assigned as material properties for the simulation. Finally, after assigning the material properties for wax, the model can be meshed. For this analysis, a 0.15 mm mesh size was chosen, and each mesh cell represents a tetrahedral solid body. The results of the FEA in the form of Von Mises’s stress are shown in Figure 8. Figure 8 shows the lateral component of Von Mises’s stress pointing toward the horizontal *Z*-axis. As can be seen from Figure 8, a maximum stress of 0.037 MPa is present in the thin film of wax, and it is responsible for thermal contraction since its direction of action is in the horizontal plane.

**Figure 9:**
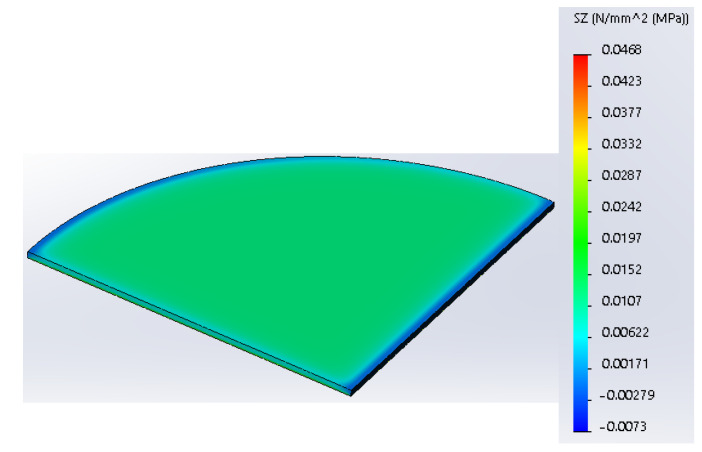
FEA results show a stress component in the lateral direction (load = 37.81 N).

Figure 9 shows the FEA results when the higher force of 37.81 N was applied, which corresponds to the plate temperature of 40 °C. This value of normal force was found by extrapolation using the line equation shown in Figure 5. This is carried out to identify the magnitude of the force at lower temperatures. As was mentioned earlier, due to some restrictions, the experiment was run at a temperature range of 90–110 °C. However, we are interested in finding the force value at the range of 40–110 °C where the material starts to respond to heating as was identified by the DSC analysis [8]. Therefore, the line equation was useful to identify the magnitude of the normal force when a wider temperature range is used. From Figure 9, it is visible that the maximum stress magnitude in the lateral direction increased to 0.046 MPa.

From found values of maximum stresses, it is evident that a 0.5 mm thick layer of deposited wax can produce a stress magnitude that is approximately 40 times less than the Yield strength (σ_y_) of the material which is equal to 1.9 MPa [22]. However, since the printed part represents a multi-layer structure, stress accumulation is inevitable. Thus, by dividing the σ_y_ by the maximum thermal stress of 0.046 MPa, we obtain a value of 41. Since the thickness of the studied layer is equal to 0.5 mm, 0.5 × 41 rounds up to 20.5 mm. Therefore, the stress accumulation up to the critical value of σ_y_ is possible when the thickness of the part reaches 20.5 mm. This is possible provided that the bonding force between the material and the print bed has been overcome. It should be noted that for rigid polymers such as ABS, much thinner parts have experienced the warpage issue [12].

**Figure 10:**
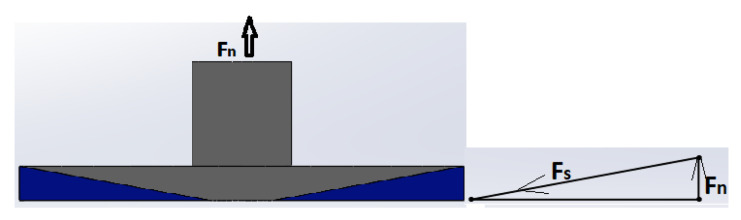
Force balance in cone plate measuring system: schematics of the cp measuring system (**left**); force balance diagram (**right**).

The results of the FEA show that the magnitude of the residual stresses is 41 times less than σ_y_ of the wax material. Therefore, for the residual stresses to reach critical levels, the 3D-printed sample should be of a thicker size. However, it has been demonstrated that warpage occurs even in thin samples [15]. Thus, it is expected that the thermal stresses must be higher than those identified by the FEA method. Moreover, it should be noted that the FEA analysis applied several assumptions. For instance, the plate geometry was considered flat, whereas the real shape of the upper plate is conical as shown in Figure 2. The analytical model can be used to account for the geometry of the measuring system. For example, Figure 10 shows the force balance in the measuring system and the wax sample under thermal stresses. As can be seen from Figure 10, the forces developed in the wax sample due to shrinkage forces F_s_, are balanced by the normal forces F_n_ developed in the measuring system of the rheometer. Since the normal forces are known from the experiment, it is not difficult to compute the thermal stresses. Here, Equation (1) can be used to identify the shrinkage forces Fs:

F_n_ = F_s_ Sin(truncate angle°) (1)

where the truncate angle is 1°. The thermal stresses σ_w_ present in the wax sample can be computed by dividing the computed F_s_ values by the area A_c_ that envelopes the inclined portion of the cone: σ_w_ = F_s_/A_c_.

**Figure 11:**
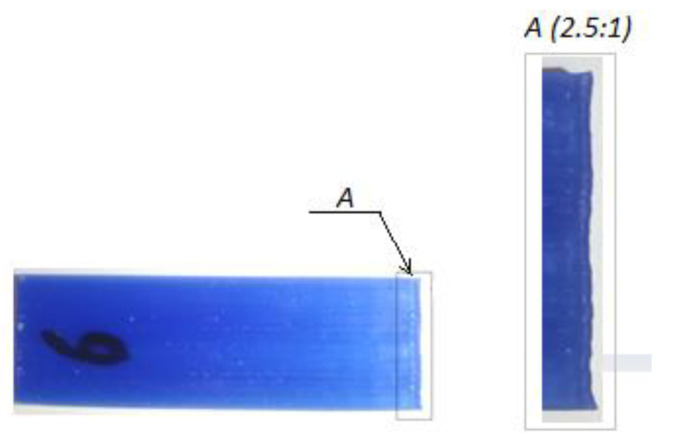
3D printed sample (**left**) with a magnified view of the wall (**right**).

Analytically found values of thermal stresses are shown in Table 3 along with findings from the FEA. From Table 3, it is clear that the calculated values of thermal stresses are much higher compared to the values found by the FEA. Thus, the plastic deformation caused by the warpage might occur in samples considerably thinner than 20.5 mm. The magnified side view of the 3D printed wax benchmark having an overall height of 20 mm is shown in Figure 11. From Figure 11, it is clear that the vertical walls at two ends of the sample have a wavy shape. That is the evidence of the occurrence of plastic deformation caused by the thermal shrinkage of the wax. By looking at the bottom of the part, it is seen that the first layers have not undergone a size contraction. However, as new layers are added, the shrinkage becomes more evident. Once the residual stresses diminish owing to the stress relaxation, newly deposited layers are restricted from shrinking by the bonding forces that hold the adjacent layers together. As the residual stresses build up more, the size contraction takes place again shaping the wavy pattern of the wall. Therefore, it can be concluded that the analytically found stress values are more accurate. Considering the stress accumulation phenomenon, the sample having several layers of thickness, each being equal to 0.5 mm, is enough to initiate the plastic deformation of the wax owing to the weak mechanical properties of the latter (σ_y_ = 1.9 MPa).

### 3.2. Results of Taguchi Analysis

A Signal-To-Noise (S/N) ratio identified by the Taguchi approach is a parameter that was used to evaluate the effect of input parameters on the warpage magnitude. “The lower the better” response was chosen because the preference is to have a low warpage value. An Equation (2) was used to find the S/N ratio as follows:

(2) $$\frac{S}{N}=-10\log_{10}\left[\frac{1}{n}\sum_{i=1}^{n}y_{i}^{2}\right]$$

where *n* is the number of repetitions and *y* is the characteristic under consideration. The main effect plot for the *S*/*N* ratio is shown in Figure 10 and the corresponding response table for *S*/*N* ratios is shown in Table 4. The main effect plots evidence that the warpage degree is mostly influenced by the nozzle speed and bed temperature. In contrast, the nozzle temperature was found to be insignificant. As can be seen from Figure 11, the maintenance of higher bed temperature is important to minimize the level of warpage. That is because the low temperature of the bed increases the temperature gradient between the molten polymer extrudate and the environment. Since warpage manifests as the detachment of the part’s outer edges from the print bed, the very first layer of the polymer must have a strong bond with the print bed [23]. This can be achieved by increasing the bed temperature.

**Figure 12:**
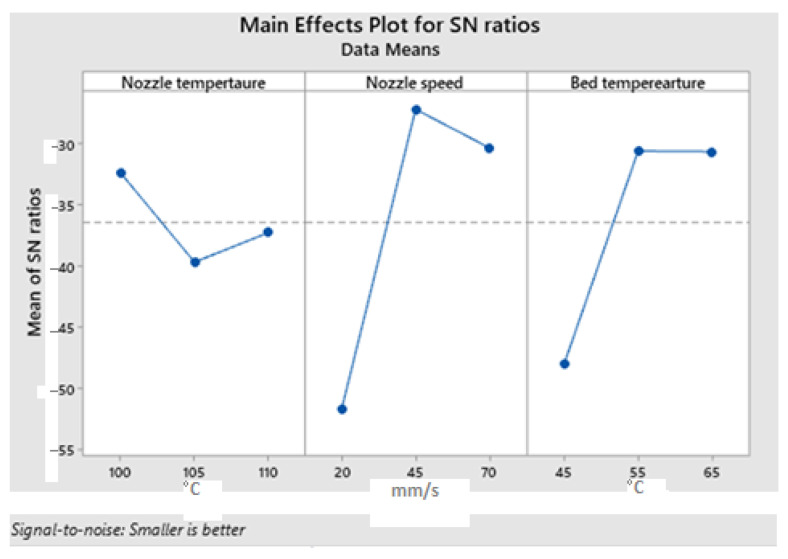
Main effects plot for S/N regarding part’s warpage magnitude.

FFF at low and high speeds is not desirable, thus, a middle value around 45 mm/s is a better choice according to the main effect plots. It has been demonstrated by Kwon et al. [24] that the influence of bed temperature on the bonding quality is more prominent than the deposition rate. However, in the case of FFF of soft polymers, it is visible that the nozzle speed has slightly higher importance. Another important aspect is the interlayer bonding. The stronger interlayer bonding is achieved by heat-driven diffusion according to Gao et al. [25]. In weakly connected layers, the level of porosity is higher, therefore, less dense parts have weak mechanical properties [26]. To increase the level of bonding, the layers of polymer should be deposited at a higher rate to ensure a strong connection to the previously placed layer. In this regard, a high printing temperature is required to ensure higher strength of the final part [27]. On the other hand, it can be inferred that the thermal stresses transfer more efficiently between layers if the layers are densely packed [27]. Therefore, 3D printing at high speeds influences the level of warpage and it is supported by the main effect plots shown in Figure 12.

Analysis of variance for S/N ratios is shown in Table 5. It can be concluded that the nozzle speed has the highest impact on the warpage magnitude with a contribution of 59%. After that, the bed temperature appears to be important too. *p* and F values of the ANOVA test at a 95% confidence range are used to observe the relevance of each process parameter. A ‘*p*’ value of less than 0.05 indicates that the parameters under consideration had a substantial impact on the experimental result. It has been determined that the most important input parameter for warpage occurrence is the printing speed with a ‘*p*’ value of 0.045. Additionally, the print bed temperature can be regarded as significant too because its *p* value is only slightly larger than 0.05.

## 4. Conclusions

The results of experimental work performed in this study have provided some insights into the warpage phenomenon that occurs during FFF manufacturing soft wax polymer. The majority of previous research in this area concerns rigid plastics such as ABS or PLA. However, the issue of warpage in soft polymers is not studied in detail. Due to the soft nature of the wax polymer, it was possible to perform a temperature sweep test on a thin film wax sample utilizing rheometer equipment. Temperature sweep test allows for the measurement of normal forces that occur as a response to the thermal shrinkage of the polymer. These forces develop within the measuring system of the rheometer as a thin sample of wax shrinks within the range of temperatures between 110 and 40 °C. The results of the thermo-mechanical experiment were used in the FEA and analytical computation to identify the shrinkage stresses that ultimately cause the warpage in parts manufactured by FFF. It was found that the stress values computed by the analytical method yielded more accurate results. With the level of thermal stress equal to 1.17 MPa, it is estimated that a part having an elongated shape with a small thickness in a horizontal direction will be subject to plastic deformation, which might manifest as a shape distortion.

In addition to the work dedicated to the estimation of shrinkage stresses, Taguchi’s design of experiments was performed to identify the significant process parameters of FFF that contribute to the warpage. It was discovered that the impact of 3D printing velocity is significant and to mitigate the warpage issue, the printing speed should be in the vicinity of 40 mm/s. Another significant factor is the print bed temperature. 3D printing using a lower temperature of the print bed is not desirable since it contributes to rapid cooling of the extrudate leading to weak adhesion of the material to the print bed. As a result, the shrinkage stresses easily overcome the bonding force between the polymer and the print bed.

## Figures and Tables

**Table 1:** Warpage issue relevant to FFF.

Material	Part Geometry	Experimental	Analytical/Numerical	Significant Factors Affecting Warpage	Ref.
Acrylonitrile Butadiene Styrene (ABS)	Rectangular plate	+	+	Length of the part, thickness Layer thickness	[11]
	Thin and thickplates	+	+	Print speed, layer thickness	[12]
	Tensile specimen	+	+	Layer thickness	[13]
	Rectangular plate	+		Bed and chamber temperature	[14]
Polylactic acid (PLA)	Thin plate	+		Layer thickness	[15]
	Rectangular block	+		Nozzle temperature	[16]
Polyphenylene sulfide (PPS)	Thin plate	+	+	Coefficient of thermal expansion	[17]
Polyurethane-based shape memory polymer	Thin plate	+	+	Bed temperature, printing speed, layer thickness	[18]

**Table 2:** Selected parameters and levels for Taguchi analysis.

Input Parameters	Symbol	Level 1	Level 2	Level 3
Nozzle temperature, °C	I	100	105	110
Nozzle speed, mm/s	II	20	45	70
Bed temperature, °C	III	45	55	65

**Table 3:** Magnitudes of stresses identified analytically.

Temperature Range, [C]	Thermal Stress, σ_w_ [MPa]	Max Stress Magnitude Found by the FEA, [MPa]
110–90	0.94	0.037
110–40	1.17	0.046

**Table 4:** Response table for the S/N ratios.

Level	Nozzle Temperature	Nozzle Speed	Bed Temperature
1	−32.36	−51.78	−48.07
2	−39.69	−27.19	−30.61
3	−37.27	−30.36	−30.65
Delta	7.33	24.59	17.47
Rank	3	1	2

**Table 5:** ANOVA S/N ratios.

Source	DF	Adj SS	Adj MS	F	*p*	Significance	Contribution
Nozzle temperature	2	83.76	41.88	1.65	0.378	Nonsignificant	4
Nozzle speed	2	1073.55	536.78	21.10	0.045	Significant	59
Bed temperature	2	608.53	304.27	11.96	0.077	Nonsignificant	32
Residual Error	2	50.89	25.44				
Total	8	1816.73					

## Data Availability

The data presented in this study are available on request from the corresponding author.
